# Retinal vein thrombosis and risk of occult cancer: A nationwide cohort study

**DOI:** 10.1002/cam4.1803

**Published:** 2018-09-27

**Authors:** Anette Tarp Hansen, Katalin Veres, Paolo Prandoni, Kasper Adelborg, Henrik Toft Sørensen

**Affiliations:** ^1^ Department of Clinical Epidemiology Aarhus University Hospital Aarhus Denmark; ^2^ Department of Clinical Biochemistry Aalborg University Hospital Aalborg Denmark; ^3^ Arianna Foundation on Anticoagulation Bologna Italy; ^4^ Department of Clinical Biochemistry Aarhus University Hospital Aarhus Denmark

**Keywords:** cohort study, neoplasm, retinal vein occlusion, risk, venous thromboembolism

## Abstract

**Background:**

Retinal vein thrombosis has in case reports been reported a clinical sign of cancer, especially hematological cancer. However, it is unclear whether retinal vein thrombosis is a marker of underlying cancer, as is the case for deep venous thrombosis and pulmonary embolism. We investigated the risk of occult cancer in patients with retinal vein thrombosis.

**Methods:**

A nationwide population‐based cohort study in Denmark on all patients diagnosed with a retinal vein thrombosis during 1994 and 2013. The main outcome measures were any cancer and site‐specific cancers <6 months, 6‐12 months, and 5 years following a retinal vein thrombosis diagnosis, as registered in the Danish Cancer Registry and the National Pathology Registry. We calculated the absolute cancer risk and computed standardized incidence ratios (SIRs) with 95% confidence intervals (CIs) for cancer within <6 months, 6‐12 months, and 5 years following a retinal vein thrombosis diagnosis.

**Results:**

Among 9589 patients with retinal vein thrombosis, we observed 1514 cancer cases. The risk of any cancer was 1.2% <6 months and 28.8% after 5 years. The <6 months SIR was 1.20 (95% CI 0.99‐1.44), 6‐12 months SIR was 1.15 (95% CI 0.94‐1.39), and the 5 years’ SIR was 1.08 (95% CI 1.03‐1.14). Stratification by age, gender, calendar year, and Charlson Comorbidity Index score did not change overall cancer risk estimates.

**Conclusion:**

Retinal vein thrombosis was not an important clinical marker for occult cancer. An extensive diagnostic cancer workup does not appear warranted for retinal vein thrombosis patients.

## 1 INTRODUCTION

Retinal vein thrombosis is the second most common cause of vision loss after diabetic retinopathy.^1^ Risk factors include atherosclerosis, advanced age, hypertension, diabetes, and hyperlipidemia,^1^, ^2^, ^3^, ^4^ which also increase the propensity for deep venous thromboembolism and pulmonary embolism.

Cancer induces a systemic pro‐coagulant state, which increases the risk of venous thromboembolism.^5^, ^6^ Thus, venous thromboembolism in the lower extremities and the lungs are markers of cancer.^7^, ^8^, ^9^, ^10^, ^11^, ^12^, ^13^ Several case reports have suggested that retinal vein thrombosis may also be a clinical sign of cancer, especially hematological cancers,^2^, ^3^, ^10^, ^14^, ^15^, ^16^, ^17^ but firm epidemiological evidence is lacking. Putative mechanisms involve direct neoplastic infiltration leading to impaired drainage and obstruction of the retinal veins, as well as dehydration and hyperviscosity caused by the cancer.^1^, ^2^, ^3^, ^16^, ^18^ To investigate these suppositions, we examined the risk of cancer following a retinal vein thrombosis compared with cancer risk in the general population.

## 2 METHODS

### 2.1 Setting

Denmark has a tax‐supported health care system ensuring equal and income‐independent free access to health care services for all residents. Individuals born in or immigrating to Denmark receive a unique Civil Personal Registration Number, enabling accurate linkage among Danish registries at the individual level.^19^ In the present study, we used data from the Danish National Patient Registry (DNPR),^20^ the Danish Cancer Registry (DCR),^21^, ^22^ and the Civil Registration System (CRS).^19^ The DNPR has recorded data on all admissions and discharges from non‐psychiatric hospitals since 1977 and from emergency rooms and outpatient clinics since 1995.^20^ Each hospital discharge or outpatient visit has been coded according to the *International Classification of Diseases*,* Eighth Revision* (ICD‐8) from 1977 until the end of 1993 and the *Tenth Revision* (ICD‐10) thereafter. The DCR contains complete prospectively collected data on all incident cases of primary cancer diagnosed in Denmark since 1943.^21^, ^22^, ^23^ DCR data include information on tumor staging according to TNM and Ann Arbor classifications.^22^ Reporting to the DCR has been mandatory for all hospital departments since 1987 and for general practitioners since 2004, ensuring nationwide completeness of its data.^21^, ^22^, ^23^ DCR data are linked to the National Pathology Registry, ensuring high validity of cancer diagnoses; 89% of diagnoses in the DCR have histopathological verification.^24^ The high quality of DCR data also is ensured by continuous manual and electronic quality control.

### 2.2 Study population

We used the DNPR and DCR to identify all patients diagnosed with retinal vein thrombosis, on an inpatient or hospital outpatient basis, between 1 January 1994 and 30 November 2013. A history of a previous cancer diagnosis was an exclusion criteria. We defined date of discharge or outpatient contact as the retinal vein thrombosis diagnosis date. We obtained information from the DNPR on comorbid conditions, including a history of deep venous thrombosis, myocardial infarction, heart failure, atrial fibrillation or flutter, chronic pulmonary disease, chronic kidney disease, diabetes mellitus, obesity, and alcoholism‐related disorders, as well as provoking factors for deep venous thrombosis within 90 days of the retinal vein thrombosis event (fracture, trauma, surgery, pregnancy). We used the Charlson Comorbidity Index to categorize patients’ comorbidity burden at the time of retinal vein thrombosis (normal: score = 0; moderate: score = 1 or 2; or severe: score ≥ 3).^25^, ^26^

### 2.3 Cancer incidence

The primary outcome was the absolute risk of all cancers recorded in the DCR. We obtained data on tumor staging at the time of diagnosis for all cancer cases according to the TNM classification and Ann Arbor classification.^22^, ^23^

### 2.4 Statistical analyses

We followed patients from the date of retinal vein thrombosis diagnosis until a diagnosis of cancer recorded in the DCR, death, emigration, or end of the study period (30 November 2013), whichever came first. We tabulated patient characteristics as described above, and for improving specificity, we calculated the proportion of patients diagnosed with retinal vein thrombosis at hospital departments of ophthalmology. We calculated absolute cancer risks using the cumulative incidence risk function, accounting for death as a competing risk.^27^ As a measure of relative risks, we furthermore calculated standardized incidence ratios (SIRs) for cancer, using indirect standardization, as the ratio of the observed number of cancer cases to the number of cancer cases expected in the general population among persons of the same age and in the same calendar period (in one‐year intervals) based on data in the DCR.^28^ We investigated the risk of cancer overall, within <6 months, 6‐12 months, and more than one year following the retinal vein thrombosis diagnosis. Analyses were stratified by age, gender, calendar year, and comorbidity burden according to Charlson comorbidity scores.^25^, ^26^ Assuming that cancers diagnosed during the first year of follow‐up were present at the time of retinal vein thrombosis diagnosis, we computed the number of patients needed to screen at the time of diagnosis in order to detect one excess cancer as the reciprocal of the excess risk.

ICD codes used in the study are provided in Table 1. All statistical analyses were performed using SAS version 9.4 (SAS Institute, Cary, NC).

**Table 1:** Diagnosis codes for study exposures, outcomes, and covariates according to the *International Classification of Diseases*,* Eighth* and* Tenth Revisions* (ICD‐8 and ICD‐10)

	ICD‐8 codes	ICD‐10 codes
Exposure		
Retinal vein thrombosis	377.08	DH348
Outcome		
Cancer	140‐209	C00‐C99
Covariates		
Diabetes	24900, 24906, 24907, 24909, 25000, 25006, 25007, 25009	E10‐E14 (except E102, E112, E142). O24 (except O24.4), G63.2, H36.0, N08.3
Atrial fibrillation or atrial flutter	42793, 42794	I48
Myocardial infarction	410	I21
Heart failure	42709, 42710, 42711, 42719, 42899, 78249	I50.0, I50.1, I50.2, I50.3, I50.8, I50.9, I11.0, I13.0, I13.2, I42.0, I42.7, I42.8, I42.9
Lower‐extremity deep venous thrombosis or pulmonary embolism	45100, 45099	I80.1‐3, I26
Chronic renal disease	249.02, 250.02, 753.10‐753.19, 582, 583, 584, 590.09, 593.20, 792	E10.2, E11.2, E14.2, N03, N05, N11.0, N14; N16, N18‐N19, N26.9, Q61.1‐Q61.4
Chronic pulmonary disease	490‐493; 515‐518	J40‐J47; J60‐J67; J68.4; J70.1; J70.3; J84.1; J92.0; J96.1; J98.2; J98.3
Obesity	277	E65‐E68
Alcoholism‐related disorders	980, 291.09‐291.99, 303.09‐303.99, 57109‐57111, 57710	F10 (except F10.0), G31.2, G62.1, G72.1, I42.6, K29.2, K86.0, Z72.1
Risk factors for venous thromboembolism		
Fracture/trauma	800‐929, 950‐959	S00‐T14
Surgery	N/A	Previous Danish classification until 1996:000000‐99960; NOMESCO classification after 1996: KA‐KQ, KX, KY
Pregnancy	630‐680	O00‐O99

The study was approved by the Danish Data Protection Agency (record number 1‐16‐02‐1‐08).

## 3 RESULTS

We followed 9589 patients diagnosed with a retinal vein thrombosis for a median of 5.1 years (25th–75th percentile: 2.2‐9.2 years; Table 2). Median age at the retinal vein thrombosis event was 70 years with equal gender distribution. Approximately 90% of patients were older than age 50 years when they experienced a retinal vein thrombosis. As expected, these patients had a higher comorbidity burden (including cardiovascular diseases) compared with patients aged below 50 years (Table 2). One‐third of the patients aged 50 years or more had undergone surgery within 90 days before the event. Nearly all retinal vein thrombosis patients (95%) were diagnosed at ophthalmologic departments.

**Table 2:** Characteristics of retinal vein thrombosis patients according to age at diagnosis, Denmark, 1997‐2013

	All retinal vein thrombosis patients	0‐50 y	50+ y
	N (%)	N (%)	N (%)
Total	9589 (100.0)	894 (100.0)	8695 (100.0)
Male sex	4714 (49.2)	495 (55.4)	4219 (48.5)
Calendar year of diagnosis			
1994‐2001	3411 (35.6)	299 (33.4)	3112 (35.8)
2002‐2009	3801 (39.6)	363 (40.6)	3438 (39.5)
2010‐2013	2377 (24.8)	232 (26.0)	2145 (24.7)
CCI comorbidity score			
Normal	6050 (63.1)	722 (80.8)	5328 (61.3)
Moderate	2868 (29.9)	149 (16.7)	2719 (31.3)
Severe	671 (7.0)	23 (2.6)	648 (7.5)
Medical history			
Myocardial infarction	552 (5.8)	11 (1.2)	541 (6.2)
Heart failure	460 (4.8)	9 (1.0)	451 (5.2)
Lower‐extremity deep venous thrombosis or pulmonary embolism	230 (2.4)	13 (1.5)	217 (2.5)
Atrial fibrillation or atrial flutter	668 (7.0)	11 (1.2)	657 (7.6)
Chronic kidney disease	217 (2.3)	25 (2.8)	192 (2.2)
Chronic pulmonary disease	725 (7.6)	25 (2.8)	700 (8.1)
Diabetes mellitus	973 (10.1)	69 (7.7)	904 (10.4)
Obesity	322 (3.4)	39 (4.4)	283 (3.3)
Alcoholism‐related disorders	189 (2.0)	30 (3.4)	159 (1.8)
Pregnancy within 90 d	7 (0.1)	7 (0.8)	0 (0)
Surgery within 90 d	3093 (32.3)	159 (17.8)	2934 (33.7)
Fracture/trauma within 90 d	253 (2.6)	22 (2.5)	231 (2.7)

CCI, Charlson Comorbidity Index.

**Figure 1:**
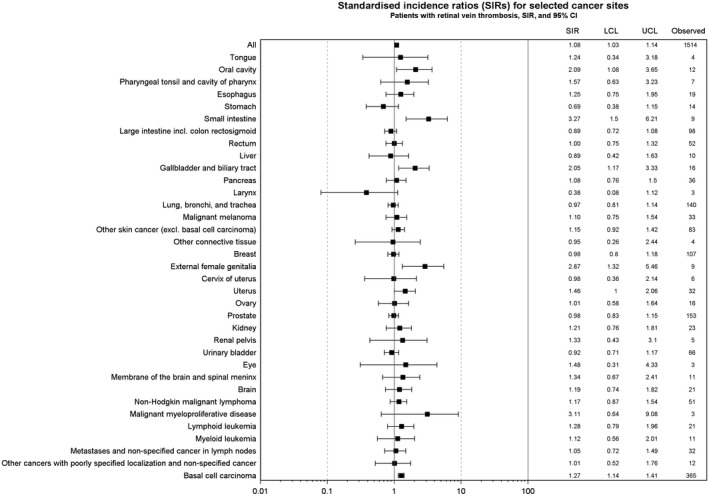
Overall and site‐specific cancer risk during entire follow‐up in patients with retinal vein thrombosis, Denmark, 1997‐2013, expressed as absolute numbers and standardized incidence rates (SIRs) with lower (LCL) upper confidence limits (UCL). Site‐specific cancers with at least three cases included in the figure

**Figure 2:**
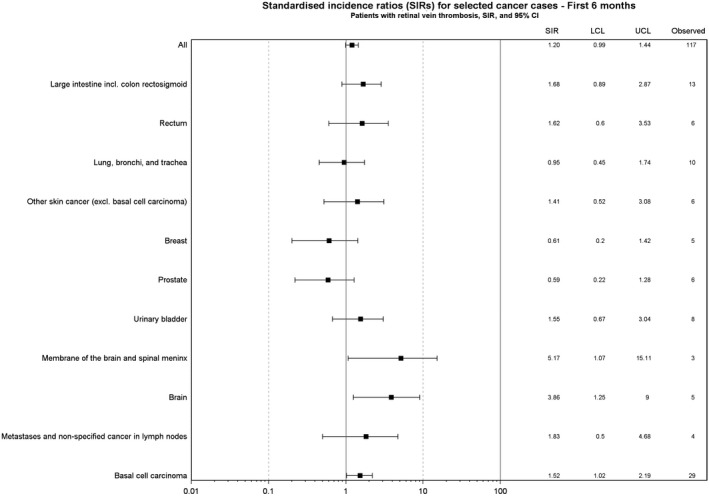
Overall and site‐specific cancer risk during first 6 mo following a retinal vein thrombosis, Denmark, 1997‐2013, expressed as absolute numbers and standardized incidence rates (SIRs) with lower (LCL) and upper confidence limits (UCL). Site‐specific cancers with at least three cases included in the figure

Normal (score = 0), moderate (score = 1‐2), or severe (score ≥ 3),

Overall, we observed 1514 cancer events (Figure 1). During overall follow‐up, we observed a 20% increase in the risk of hematological cancer among patients with retinal vein thrombosis (overall SIR 1.20, 95% CI 0.97‐1.48, data not shown). The <6‐month risk of any cancer was 1.2% (95% CI 1.0%‐1.5%), increasing to 2.4% (95% CI 2.1%‐2.7%) one year after retinal vein thrombosis, and 28.8% (95% CI 26.9%‐30.8%) >5 years (end of follow‐up). The <6 months’ SIR was 1.20 (95% CI 0.99‐1.44; Figure 2) and remained unchanged 6‐12 months following retinal vein thrombosis (SIR 1.15, 95% CI 0.94‐1.39), >1 year after the event (SIR 1.07, 95% CI, 1.01‐1.13), and ≥5 years after the event (SIR 1.08, 95% CI 1.03‐1.14).

**Figure 3:**
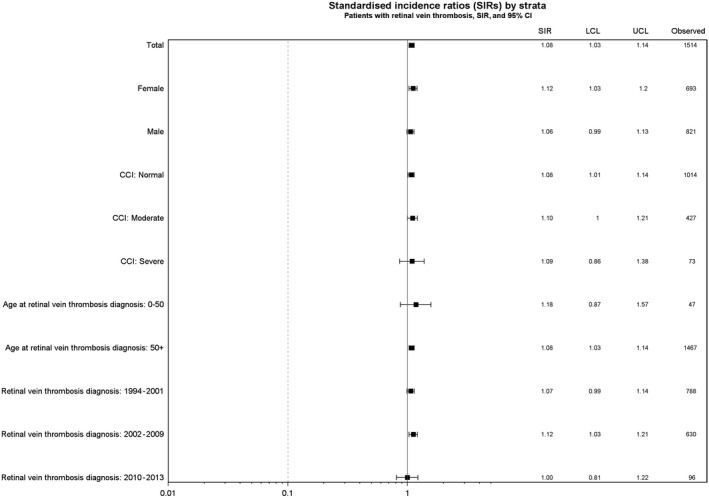
Overall cancer risk during entire follow‐up in patients with retinal vein thrombosis, Denmark, 1997‐2013, expressed as absolute numbers and standardized incidence rates (SIRs) with lower (LCL) upper confidence limits (UCL).Stratified on age and calendar year at retinal vein thrombosis, gender, and Charlson Comobidity Index (CCI)

Among patients with retinal vein thrombosis, 53% of cancers were localized tumors, 12% were tumors with regional spread, 15% were tumors with distant metastases, and 20% had missing data on tumor staging. The overall SIR estimates were almost similar in subgroups of patients, according to age, gender, calendar year, and comorbidity burden, measured by the Charlson comorbidity score (Figure 3). Based on 33 excess cancers detected during a follow‐up time of 8969 person‐years (corresponding to the first year of follow‐up), the number of patients with retinal vein thrombosis needed to screen to detect one excess cancer per year was 271 for any cancer.

## 4 DISCUSSION

In this nationwide population‐based cohort study, the absolute cancer risk within six months following a retinal vein thrombosis was low and only slightly increased compared to the general population. Thus, retinal vein thrombosis was not an important clinical marker for occult cancer. Interestingly, the relative cancer risk beyond the first year following retinal vein thromboses was comparable with the cancer risk reported for deep venous thrombosis.^8^ This is probably explained by the comorbidity burden or common risk factors. Previous case reports suggesting an association between retinal vein thrombosis and hematological cancers could not be confirmed.^2^, ^3^, ^10^, ^14^, ^15^, ^16^, ^17^

Although deep venous thrombosis and pulmonary embolism are strong markers for occult cancer,^7^, ^8^, ^9^, ^11^, ^12^, ^13^ this was not observed for retinal vein thrombosis. This suggests that different underlying mechanisms are likely to be involved in the pathophysiology of venous thrombosis at different vascular sites. Disturbances in one or more of the components of the Virchow's triad (stasis of blood flow, vessel wall injury, and hypercoagulability) usually explain deep venous thrombosis and pulmonary embolism. Activation of the coagulation cascade, a systemic hypercoagulability, is essential for the venous clot formation observed in the larger veins^11^, ^13^ and may be caused by cancer. In contrast, it seems likely that most cases of retinal vein thrombosis in our study were caused by shared risk factors for atherosclerosis, *for example,* stiffness in adjacent arteriosclerotic arteries leading to turbulence and retinal thrombus formation.^1^, ^2^, ^3^, ^4^ This was supported by the high prevalence of arteriosclerosis we observed, especially in the older age groups. By contrast, the subgroup of young patients had a low comorbidity burden and, not surprisingly, had an increased cancer risk within the first year following a retinal vein thrombosis. Other etiologies may have played a role for their thrombosis, for example, underlying cancer. Nevertheless, the estimates for this subgroup were based on few cancer cases with rather imprecise risk estimates. The present study was overall a negative study. Furthermore, we reported no *P*‐values and made no significance testing. Therefore, adjustments for multiple comparisons are not recommended.^29^, ^30^ However, in the present study on cancer risk with multiple comparisons, we cannot rule out by chance findings.

We based our study on prospectively collected nationwide data with complete population coverage in the setting of a universal tax‐supported health care system, which likely eliminated selection and recall bias. Confounding is generally not a problem in the reporting of absolute risks. As regards the relative SIR estimates, stratification by possible confounders did not substantially change the SIRs. The validity of the retinal vein thrombosis in the DNPR is assumed to be high, because nearly all retinal vein thrombosis patients were diagnosed at ophthalmologic departments. Nevertheless, we cannot rule out misclassification of some of the retinal vein thrombotic events. The validity of cancer diagnoses in the DCR is high.^21^, ^22^, ^23^

In conclusion, the absolute risk of occult cancer in retinal vein thrombosis was low, as reflected in the high number needed to screen. An extensive diagnostic cancer workup does not appear warranted for retinal vein thrombosis patients. The clinical impact of these negative findings is important. Furthermore, the results of our study may contribute to our understanding of the mechanisms of carcinogenesis. The occult cancer‐associated hypercoagulabilty contributing to an increased risk of venous thromboembolism does not seem to cause retinal vein thrombosis.^11^

## CONFLICT OF INTEREST

The authors declare no conflict of interests.
